# High efficiency electrocatalyst of LaCr_0.5_Fe_0.5_O_3_ nanoparticles on oxygen-evolution reaction

**DOI:** 10.1038/s41598-020-70283-9

**Published:** 2020-08-07

**Authors:** Xiaoping Gao, Zhimin Sun, Jiaqi Ran, Jufu Li, Jingyan Zhang, Daqiang Gao

**Affiliations:** 1 Key Laboratory of Sensor and Sensing Technology, Gansu Academy of Sciences, Lanzhou, 730000 Gansu China; 2 Lanzhou Resources and Environment Voc-tech College, Lanzhou, 730000 Gansu China; 3 Key Laboratory for Magnetism and Magnetic Materials of MOE, Key Laboratory of Special Function Materials and Structure Design of MOE, Lanzhou University, Lanzhou, 730000 China

**Keywords:** Chemistry, Energy science and technology

## Abstract

Due to the multistep proton-coupled electron transfer, it remains a huge challenge to accelerate the kinetics of oxygen evolution reaction (OER). Here, we demonstrate that perovskite-type LaCr_0.5_Fe_0.5_O_3_ nanoparticles can be used as highly active and stable OER electrocatalysts, where it shows a low overpotential of 390 mV at 10 mA/cm^2^, a small Tafel slope of 114.4 mV/dec and excellent stability with slight current decrease after 20 h, superior than that of their individual counterparts (LaFeO_3_ and LaCrO_3_). This finding confirms that the present hybrid material would be an effective means to electrocatalyst for catalyzing OER.

## Introduction

It is very urgent that develop low cost, high stability and efficient energy convert and storage system (eg. metal-air batteries^1^, fuel cells^2^ and super capacitors^3^) for clean energy. Oxygen evolution reaction (OER) is the important step for many energy storage systems^4–6^, where noble metal-based (eg. IrO_2_/RuO_2_) catalysts are generally used as the most active catalysts to accelerate the kinetics of OER. However, it is difficult to meet the needs of commercialization, due to its scarcity and expensive of these precious metals^7^. Therefore, it is inevitable and great interest to explore the efficient and low-cost alternative non-precious electrocatalysts.

Recently, the stable structural, controllable electronic states^8^ and relatively high ionic/electronic conductivity make oxide perovskite electrocatalysts popular. In particular, as the most important parent oxides in the family of perovskite, LaFeO_3_ has been widely reported as efficient electrocatalyst. Unfortunately, calculations^9,10^ and experimental measurements^11^ results proved that individual LaFeO_3_ perovskite has poor oxygen reduction reactions (ORR) and oxygen evolution reaction (OER) activity in alkaline electrolyte. As a consequence, many attempts have been used to optimize the electronic structure and further optimize the ORR/OER activity of LaFeO_3_. For instance, Wang et al. used phosphorus doping strategy to optimize the OER and ORR electrocatalytic activity of LaFeO_3-δ_ perovskite^12^. Copper and zinc co-doped LaFeO_3_ perovskite oxides with enhanced electrocatalytic activity were also reported by Mahmoud Omari^13^. Schon et al. reported the highest catalytic activity for CO oxidation in LaFe_0.8_Cu_0.2_O_3_^14^. Furthermore, the OER activity of LaFeO_3_ was enhanced through coating an amorphous cobalt-phosphate^15^. Based on the conclusions reported in the above articles, we can see that the A or B ions in the perovskite oxide (ABO_3_) are partially replaced by other ions can cause changes in oxygen vacancies, ion valence and structural distortions. These can also lead to the improvement of its catalytic performance. Generally speaking, the stability and electrocatalytic activity of the perovskite oxide are more affected by the B-site cation^16^. Therefore, the method of replacing the B-site cation by incorporating some heteroatoms is an effective strategy to promote the electrocatalytic activity.

Herein, double perovskite LaCr_x_Fe_1−x_O_3_ with enhanced OER activity is reported, where the optimized sample of LaCr_0.5_Fe_0.5_O_3_ displays the lowest overpotential of 390 mV at 10 mA/cm^2^, Tafel slope of 114.4 mV/dec and long-term durability (20 h). We also through the electrocatalytic test and the first principle calculations results prove that the excellent performance of LaCr_0.5_Fe_0.5_O_3_ are raised from the improved conductivity, tailored electronic structure and strong hybridization between oxygen and imetallic atoms.

## Results and discussion

**Figure 1:**
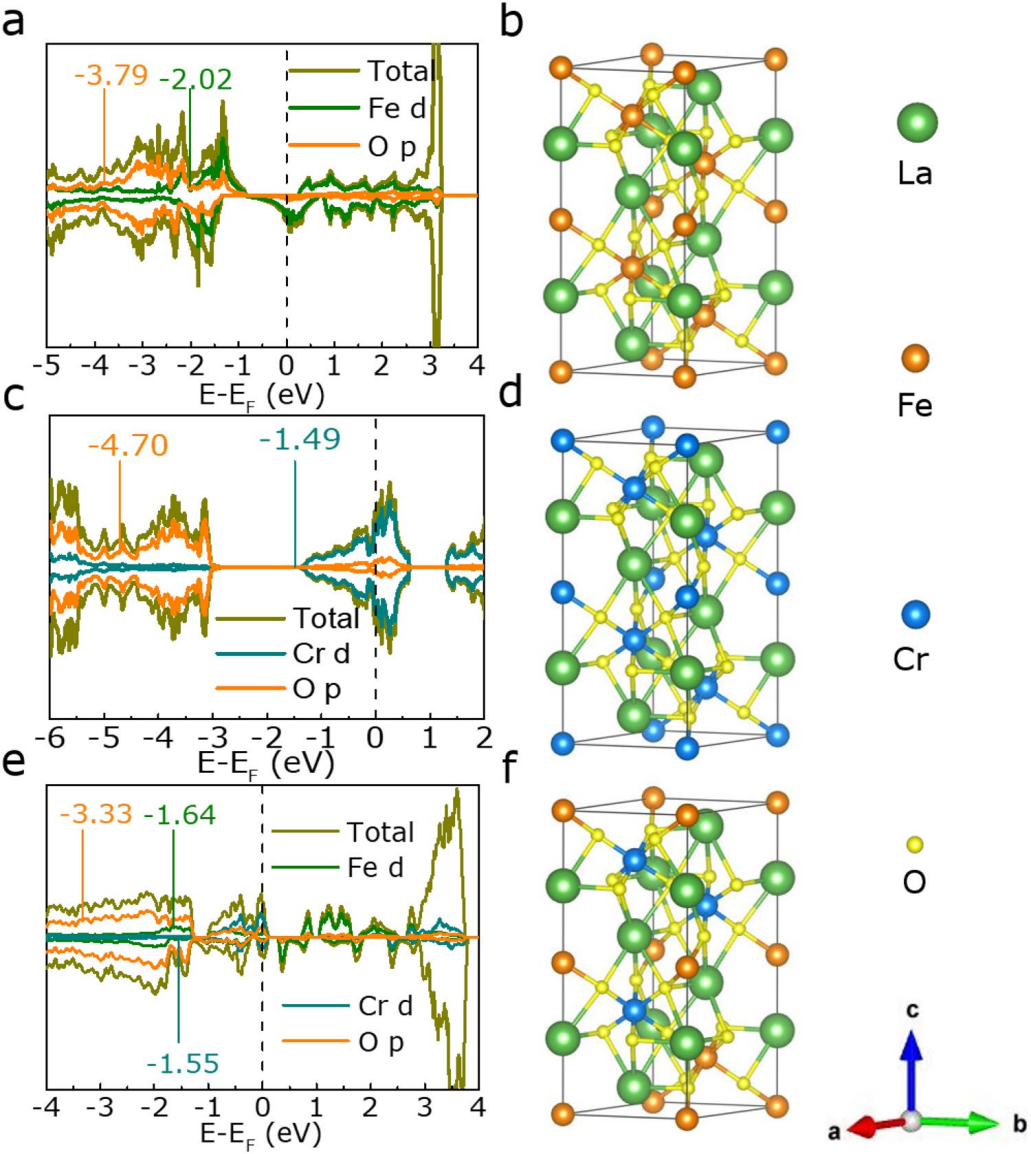
The DOS diagram of (**a**) LaFeO_3_, (**c**) LaCrO_3_ and (**e**) LaCr_0.5_Fe_0.5_O_3_. The atomic structure optimization diagram of (**b**) LaFeO_3_, (**d**) LaCrO_3_ and (**e**) LaCr_0.5_Fe_0.5_O_3_.

Since the OER electrocatalytic activity of the catalyst is highly correlated with its electronic structure, we therefore first verify this relationship through first principle calculation. Figure 1 shows the calculated the density of states (DOS) results of LaFeO_3_, LaCr_0.5_Fe_0.5_O_3_ and LaCrO_3_ models. They all exhibit the full electron occupied states at the Fermi level, revealing the metal-like state of LaFeO_3_, LaCr_0.5_Fe_0.5_O_3_ and LaCrO_3_. For LaCr_0.5_Fe_0.5_O_3_ case, there are more electron occupied states at the Fermi level, ascribing the strong hybridization of Fed/Cr-d/O-p orbits. Furthermore, it is well-known that the adsorbate–metal interactions can be predicted by the energy difference between d-band center and Fermi level (∆*E*_O_)^17,18^. Based on the d-band center model, the smaller value of ∆*E*_O_, the stronger bond between the metal atom and the adsorbed oxygen-containing substance, which means the higher intrinsic OER activity^19,20^. Apparently, our calculation results indicate that the LaCr_0.5_Fe_0.5_O_3_ exhibits the smallest ∆*E*_O_ value [O(− 3.33 eV), Fe(− 1.64 eV) ,Cr(− 1.55 eV)] compared with LaFeO_3_ [O(− 3.79 eV), Fe(− 2.02 eV)] and LaCrO_3_ [O(− 4.70 eV), Cr(− 1.49 eV)], revealing the OER activity and electron can be significantly promoted through the strongest binding between surface Cr/Fe atoms and the adsorbed oxygenated species upon electrolysis.

**Figure 2:**
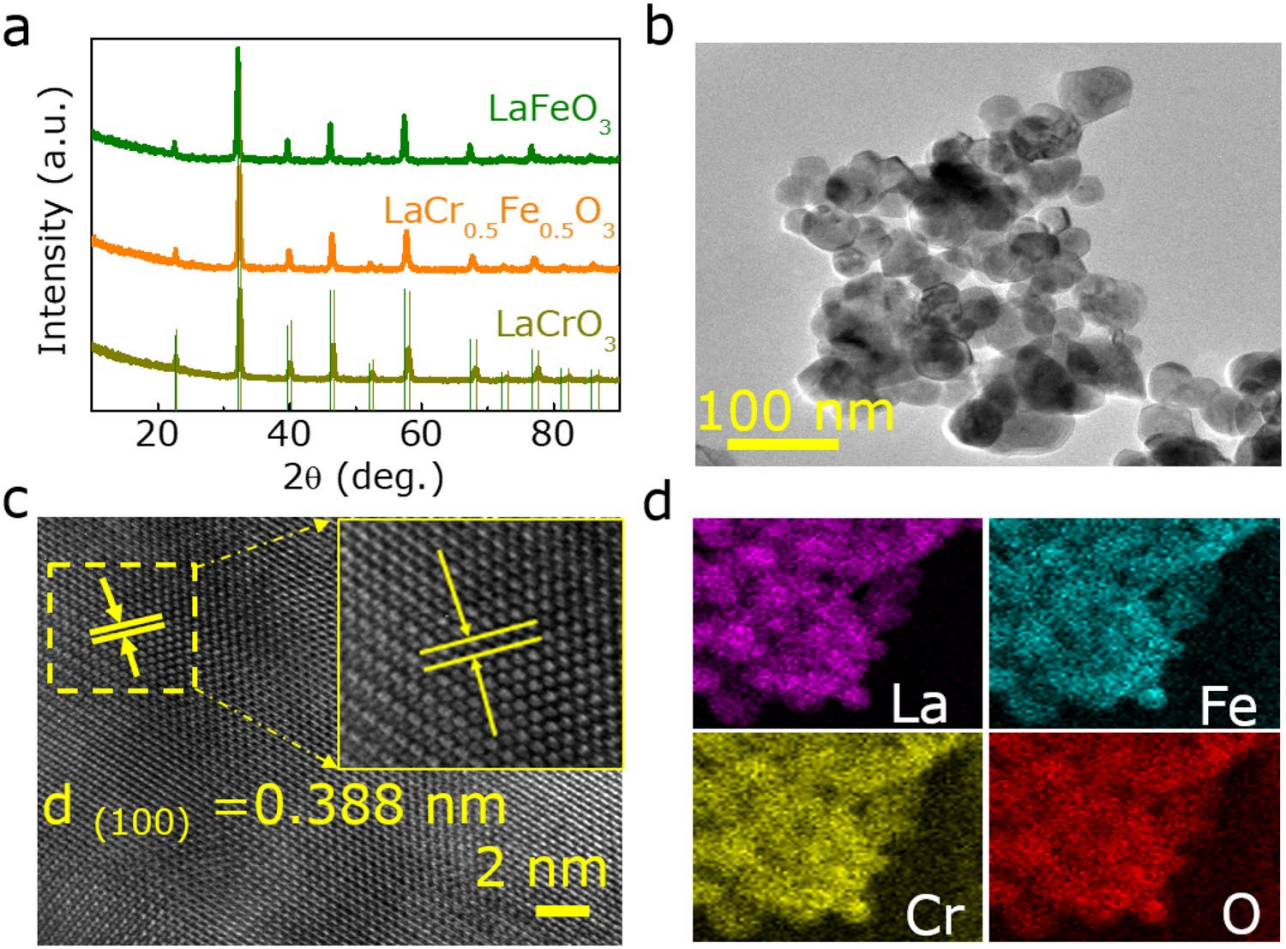
(**a**) XRD patterns of LaFeO_3_, LaCr_0.5_Fe_0.5_O_3_ and LaCrO_3_. (**b**) TEM images of LaCr_0.5_Fe_0.5_O_3_. (**c**) HRTEM image of LaCr_0.5_Fe_0.5_O_3_. (**d**) Corresponding EDX element mapping images of LaCr_0.5_Fe_0.5_O_3_.

Figure 2a and Figure S1 (Supporting Information) show the X-ray diffraction (XRD) patterns of as-synthesized LaCr_x_Fe_1−x_O_3_ (x = 0, 0.2, 0.5, 0.7, 0.9, 1, respectively) perovskite oxides. The diffraction peak of LaFeO_3_ (x = 0) corresponds to the standard cubic spinel LaFeO_3_ (PDF# 75-0541). Nevertheless, as the concentration of Cr increased, the characteristic peaks gradually shifts to the body-centered tetragonal spinel structure of LaCrO_3_ (PDF #75-0441)^21^. The morphology of the as-prepared LaCr_0.5_Fe_0.5_O_3_ was examined by scanning electron microscopy (SEM) and transmission electron microscopy (TEM) as presented in Figure S2 (Supporting Information) and Fig. 2b, respectively. The morphology of LaCr_0.5_Fe_0.5_O_3_ displays some pore structures formed by aggregated or randomly stacked irregularly shaped particles. Noting that all the samples show the same particles morphology as revealed in Figures S2, S3 (Supporting Information). The high-resolution TEM (HRTEM) was carried out to further characterize the structures of LaCr_0.5_Fe_0.5_O_3_. As shown in Fig. 2c, the obvious lattice fringe of 0.388 nm matched with the (100) plane. Further, as revealed by the high-angle annular dark-field scanning TEM-EDS mapping (Fig. 2d), the element distributions are highly even in LaCr_0.5_Fe_0.5_O_3_.

**Figure 3:**
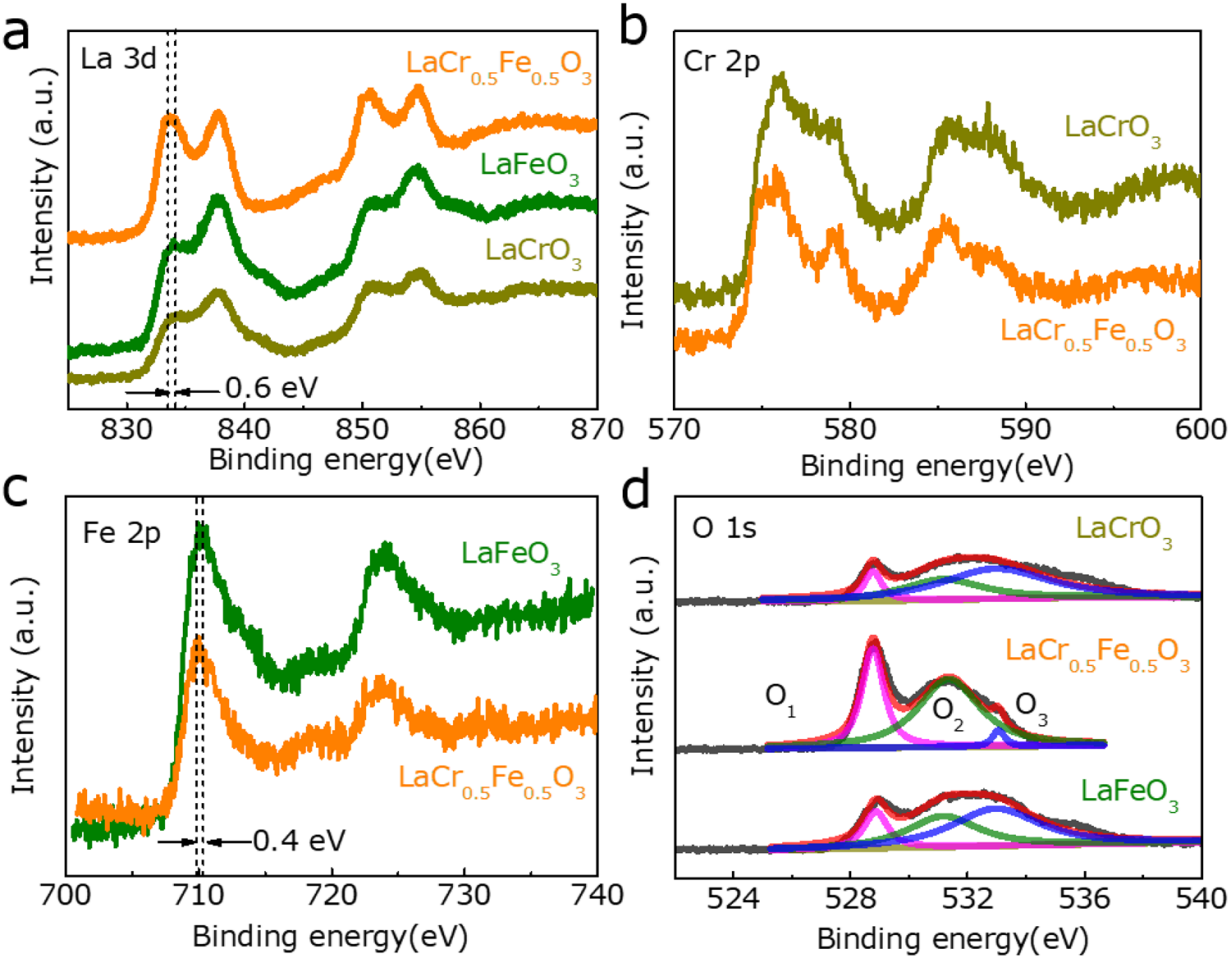
XPS spectrum of (**a**) La 3d, (**b**) Cr 2p, (**c**) Fe 2p and (**d**) O 1 s.

The oxidation state and composition of the electracatalysts were detected via X-ray photoelectron spectroscopy (XPS). Figure 3a exhibits La 3d spectra of LaFeO_3_, LaCr_0.5_Fe_0.5_O_3_ and LaCrO_3_, which are deconvoluted into two different characteristic peaks of La 3d_3/2_ (850.4 and 854.7 eV) and La 3d_5/2_ (833.5 and 837.5 eV)^22^. Noting that the peak positions of LaCr_0.5_Fe_0.5_O_3_ have a slight shift to low energy compared with LaFeO_3_ and LaCrO_3_, revealing that there are valence change or electron transfer in LaCr_0.5_Fe_0.5_O_3_. The high resolution spectrums of Cr 2p_1/2_ and Cr 2p_3/2_ peaks are consistent with 586.2 and 577.0 eV, respectively^23^. As can be seen in Fig. 3b, the peak at 577 eV of Cr 2p_3/2_ can be assigned to Cr^3+^, and as the literature reports that the catalytic activity will be promoted by Cr^3+^ of the high-valence Cr species^24^. As shown in Fig. 3c, the peaks at 709.9 and 723.6 eV were assigned to Fe 2p_3/2_ and Fe 2p_1/2_, respectively^25^. Compared to the LaFeO_3_, the Fe 2p_3/2_ peaks of LaCr_0.5_Fe_0.5_O_3_ shift from 709.9 eV to 710.2 eV. The O 1 s spectrum illustrated in Fig. 3d shows three peaks that are matched with lattice oxygen (O_1_, 529.8 eV), defect oxygen (O_2_, 531.5 eV) and surface absorbed oxygen (O_3_, 533.0 eV), respectively^26^. Furthermore, the result indicates that LaCr_0.5_Fe_0.5_O_3_ possesses higher ratio of oxygen defects (48.4%) than other samples (LaCrO_3_-31.3%, LaFeO_3_-40.7%), indicating that Cr doping can introduce more oxygen defects, which will enhance the interaction of hybrids and absorbed oxygen-containing species^27^.

**Figure 4:**
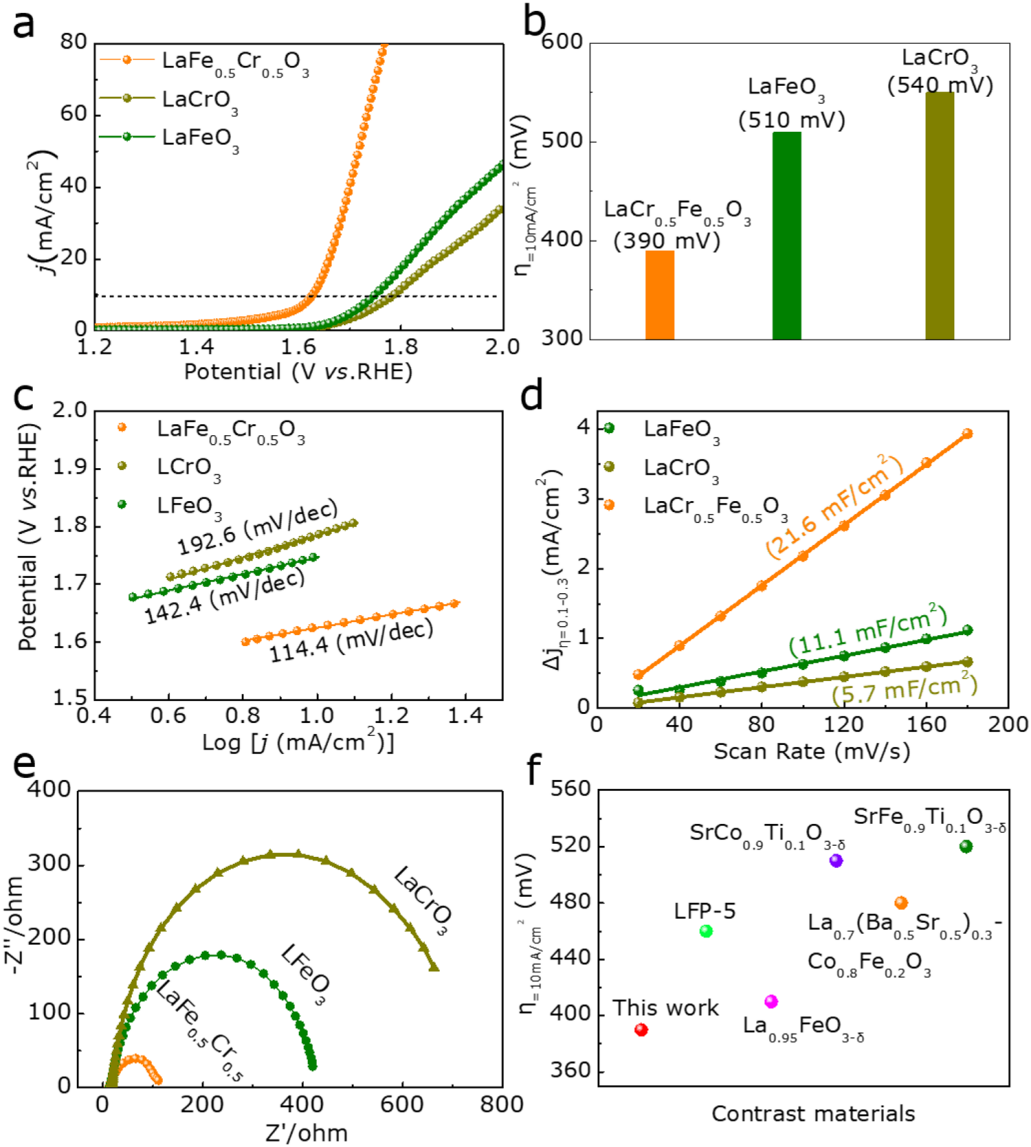
(**a**) The polarization curves, (**b**) overpotential at 10 mA/cm^2^ and (**c**) Tafel plots of LaFeO_3_, LaCr_0.5_Fe_0.5_O_3_ and LaCrO_3_ for OER. (**d**) The CV curcalculated Cdl of all samples (**e**) EIS Nyquist plots of all samples. (**f**) Overpotential (η) of previously reported material for OER at 10 mA/cm^2^.

As displayed in Table S1 and Figures S4, S6 (Supporting Information), the ration of *x* in LaCr_x_Fe_1−x_O_3_ (x = 0, 0.2, 0.5, 0.7, 0.9, 1, respectively) can tune the electrocatalytic activity of the electrocatalysts and the optimized *x* is about 0.5. Thus, the linear sweep voltammetry (LSV) of LaCr_0.5_Fe_0.5_O_3_ reveals an OER overpotential at 10 mA/cm^2^ of 390 mV, which is smaller than 510 mV of LaFeO_3_ and 550 mV of LaCrO_3_ (Fig. 4a,b). Besides, as shown in Fig. 4c, LaCr_0.5_Fe_0.5_O_3_ owns the lowest Tafel slope (114.4 mV/dec) among LaFeO_3_ (142.4 mV/dec) and LaCrO_3_ (192.6 mV/dec), respectively. In addition, the cyclic voltammetry (CV) curve (Figure S5, Supporting Information) illustrates that C_dl_ (Fig. 4d) of LaCr_0.5_Fe_0.5_O_3_ is about 21.6 mF/cm^2^, larger than LaFeO_3_ (11.1 mF/cm^2^) and LaCrO_3_ (5.7 mF/cm^2^). To our knowledge, C_dl_ is proportional to electrocatalytically active surface areas (ECSA) of electrocatalysts^28^, and the largest C_dl_ of LaCr_0.5_Fe_0.5_O_3_ indicates it possesses the largest ESCA. In addition, we also can get the Charge transfer resistance by electrochemial impedance spectra (EIS)^29^ in Fig. 4e. It can be seen that the charge transfer resistance of LaCr_0.5_Fe_0.5_O_3_ is smaller than that of LaFeO_3_ and LaCrO_3_, demonstrating that LaCr_0.5_Fe_0.5_O_3_ owns fastest OER rates and the optimal charge transfer capability. Further, as summarized in Fig. 4f, the LaCr_0.5_Fe_0.5_O_3_ shows better electrocatalytic activity than some recent reported electrocatalysts, such as LFP-5^12^, La_0.95_FeO_3-δ_^30^, SrCo_0.9_Ti_0.1_O_3-δ_^31^, La_0.7_ (Ba_0.5_Sr_0.5_)_0.3_Co_0.8_Fe_0.2_O_3_^32^, and SrFe_0.9_Ti_0.1_O_3-δ_^31^.

**Figure 5:**
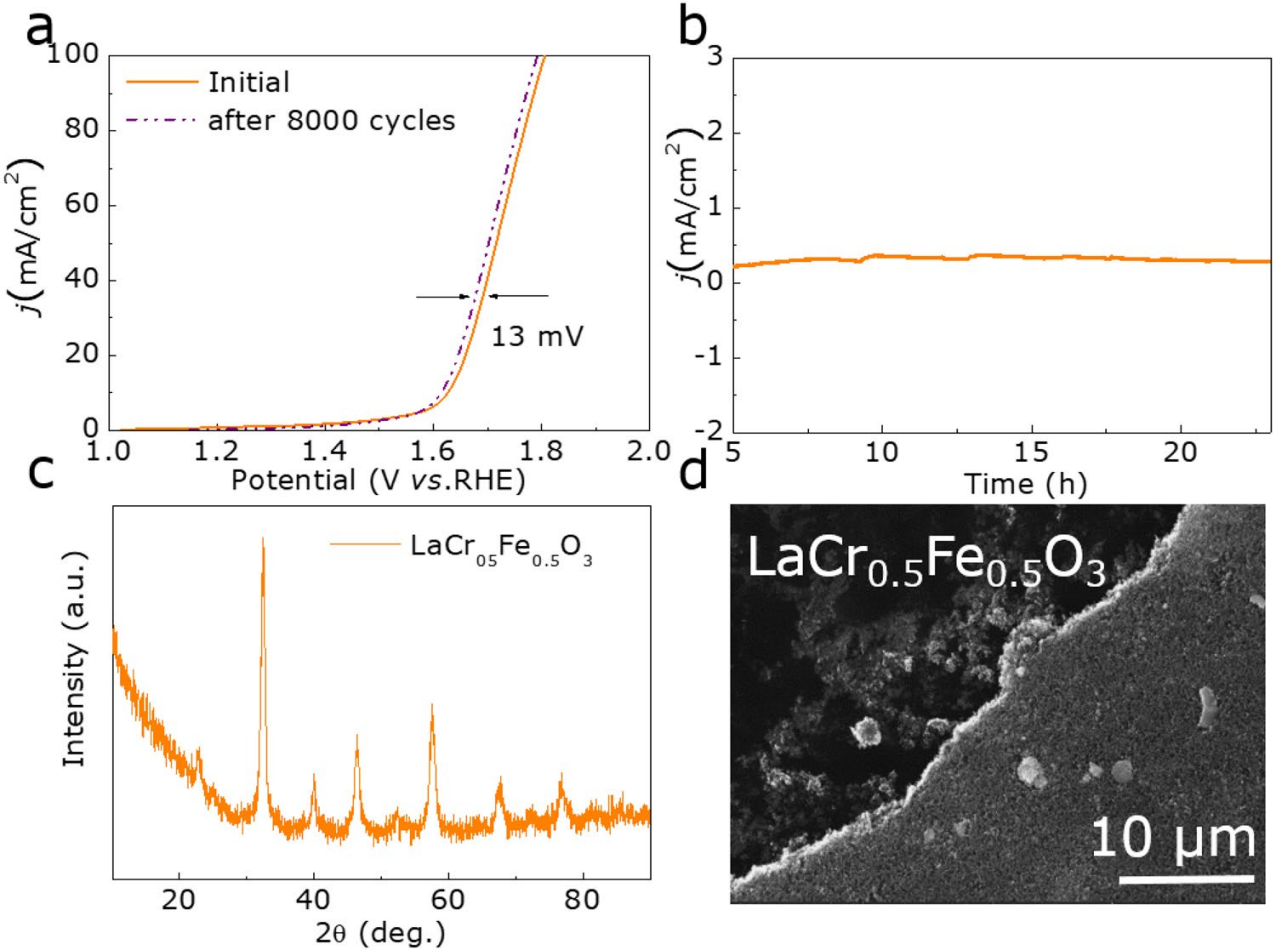
(**a**) LSV curves of LaCr_0.4_Fe_0.6_O_3_ catalyst before and after1000 cycles. (**b**) The chronoamperometry test of LaCr_0.5_Fe_0.5_O_3_. (**c**) The XRD pattern of LaCr_0.5_Fe_0.5_O_3_ after OER test. (**d**) The SEM image of LaCr_0.5_Fe_0.5_O_3_ after OER test.

At the same time, the excellent stability of the electrocatalyst can also be proved by the following tests. Figure 5a exhibits that the change of the overpotential (η) for LaCr_0.5_Fe_0.5_O_3_ is about 13 mV after 8,000 cycles under 35 mA/cm^2^. Moreover, the chronoamperometry test also can be carried out to prove the excellent stability of the LaCr_0.5_Fe_0.5_O_3_. It can be acquired from Fig. 5b and Figure S7 (Supporting Information) that the initial activity of LaCr_0.5_Fe_0.5_O_3_ remains almost unchanged after 20 h, demonstrating the outstanding stability of LaCr_0.5_Fe_0.5_O_3_ for OER. In addition, the XRD pattern (Fig. 5c), SEM image (Fig. 5d),TEM image (Figure S6a, Supporting Information) and HRTEM image (Figure S6b, Supporting Information) show that the structure is basically consistent with that before the OER test, confirming the LaCr_0.5_Fe_0.5_O_3_ has good structural stability. In light of all these findings, it is corroborated that LaCr_0.5_Fe_0.5_O_3_ possesses excellent electrocatalytic stability.

## Conclusions

In summary, theoretical calculations demonstrate that LaCr_0.5_Fe_0.5_O_3_ possesses the stronger bond between the metal atom and the adsorbed oxygen-containing substance, and greater electrical conductivity for electron transport, thus having better OER electrocatalysis active. Experimentally, electrocatalytic activity of LaCr_x_Fe_1−x_O_3_ (x = 0, 0.2, 0.5, 0.7, 0.9, 1, respectively) perovskites are studied, where the sample LaCr_0.5_Fe_0.5_O_3_ shows highly active (lower overpotential and larger ESCA) and excellent stability (structural and test stability). These results prove that doping the B-site perovskite oxide is an effective strategy to conceive more efficient and stable OER electrocatalysts.

## Experimental section

### Preparation of LaCr_x_Fe_1−x_O_3_

The catalysts of LaCr_x_Fe_1−x_O_3_ (*x* represent the amount of Cr incorporated ration in the sample, x = 0, 0.2, 0.5, 0.7, 0.9, 1) nanostructures were prepared by conventional sol–gel method. First of all, (0.005 mol) La(NO_3_)_2_.6H_2_O, (0.005*1−x mol) Fe(NO_3_)_3_.9H_2_O, (0.005*x mol) Cr(NO_3_)_3_.9H_2_O and (0.01 mol) C_6_H_8_O_7_ were added in 30 mL solution (V_H2O_:V_C2H6O_ = 1:2) with 10 min stirring. Next, the uniform mixture was dried in an oven (90 °C for 24 h). Subsequently, the precursor was annealed under air atmosphere at 600 °C for 5 h with a 2 °C min^−1^ rate of heating. When the value of x is 0.5, we name it LaCr_0.5_Fe_0.5_O_3_. By analogy we have prepared LaFeO_3_, LaCr_0.2_Fe_0.8_O_3_, LaCr_0.7_Fe_0.3_O_3_, LaCr_0.9_Fe_0.1_O_3_, LaCrO_3_, respectively.

## Supplementary information

Supplementary Information
