# Supplementary material for: High efficiency electrocatalyst of LaCr0.5Fe0.5O3 nanoparticles on oxygen-evolution reaction
Source: Sci Rep. 2020 Aug 7;10:13395. doi: 10.1038/s41598-020-70283-9 (PMC7414862; doi:10.1038/s41598-020-70283-9)
Supplement: Supplementary file 1 — Supplementary Information [file 41598_2020_70283_MOESM1_ESM.docx]

**Supporting Information**

High efficiency electrocatalyst of LaCr_0.5_Fe_0.5_O_3_ nanoparticles on Oxygen-Evolution Reaction

Xiaoping Gao,^a^ Zhimin Sun ^b*^, Jiaqi Ran, ^c^ Jufu Li, ^c^ JingyanZhang, ^c^ DaqiangGao**^c^**

^a^Key Laboratory of Sensor and Sensing Technology, Gansu Academy of Sciences, Lanzhou 730000, Gansu, China

^b^ Lanzhou resources&environment voc-tech college, Lanzhou 730000, Gansu, China

**^c^**Key Laboratory for Magnetism and Magnetic Materials of MOE, Key Laboratory of Special Function Materials and Structure Design of MOE, Lanzhou University, Lanzhou 730000, P. R. China

^*^corresponding author: zminsun@163.com

**Material characterizations**

The x-ray diffraction (XRD) in a Philips/X, Pert PRO diffractometer (Cu Ka radiation) was employed to analyze the crystal structure of the samples. Morphologies and high-resolution images are studied by scanning electron microscopy (Apreo S) and transmission electron microscopy (Tecnai G2 F30, FEI). Elemental mappings were measured by EDX, which attached to the TEM (Tecnai G2 F30, FEI). An x-ray photoelectron spectroscopy (Kratos AXIS Ultra) could be employed to identify the elementary composition of the samples and the bonding characteristics.

**Electrochemical measurements**

Using an electrochemical workstation (CHI 660E) for electrochemical measurements with a conventional three-electrode at room temperature. The glassy carbon electrode is used as a working electrode, Ag/AgCl (or Hg/HgO) is used as a reference electrode and platinum foil is used as a counter electrode, respectively. In this work, the working electrode is made as follows: 20 mg of carbon and 20 mg of the catalysts were sonicated with a moderate amount of petroleum ether for 3 h and dried, then 6 mg of the mixture was added into 1470 μl N, N dimethylformamide (DMF) solution with 30 μl Nafion-117 (the binder), and ultrasonicated for 3 h to form an evenly dispersed catalyst ink. Finally, loading the aforementioned ink onto working electrode with 9 μl, and then dried the working electrode at room temperature. The tested potentials were all corrected by RHE and IR in light of the Nernst equation {E_RHE_=E_Ag/AgCl_+0.197+0.059*pH}. All the tests of OER was tested in a 1 M KOH solution at 25 °C.

Calculated electrochemical active surface area

$A_{ECSA}^{LCO} = \frac{5.7\times1000 \mu F {cm}^{-2}}{40 \mu F {cm}^{-2} per {cm}_{ECSA}^{2}}=142.5 {cm}_{ECSA}^{2}$ (1)

$A_{ECSA}^{S_{4.23\%}-LCO} = \frac{11.1\times1000 \mu F {cm}^{-2}}{40 \mu F {cm}^{-2} per {cm}_{ECSA}^{2}}=277.5{cm}_{ECSA}^{2}$ (2)

$A_{ECSA}^{S_{5.84\%}-LCO} = \frac{21.6\times1000 \mu F {cm}^{-2}}{40 \mu F {cm}^{-2} per {cm}_{ECSA}^{2}}=540 {cm}_{ECSA}^{2}$ (3)

**Calculation method**

All the calculations based on the density functional theory (DFT) were carried out by using the Vienna ab initio simulation package (VASP)^[1-2]^. The electron-ion interactions were considered via the Projector augmented wave (PAW) method ^[3]^. Atomic structures relaxation, considering the exchange and correlation effects, was analyzed by the generalized gradient approximation with Perdew-Burke-Ernzerhof (GGA-PBE) functional ^[4-5]^. Through all the calculations, the cut-off energy was selected as 400 eV and a 7×7×7 Monkhorst-Pack K-points were considered for the Brillouin-zone integration ^[6]^. Besides, the energy convergence threshold was set as 10^−5^ eV and ionic relaxations were conducted until all force components became <0.02 eV Å^-1^. Herein, optimized geometry cells of LaCrO_3_ and LaFeO_3_ were used in our calculations. Moreover, there Cr dopants were introduced to replace the equivalent Fe atoms in the cell of LaFeO_3_, forming the Cr-LaFeO_3_ system, whose Fe, Cr ratios are close to that of our experimental samples. And their density of states (DOS) and charge distributions were fully studied.


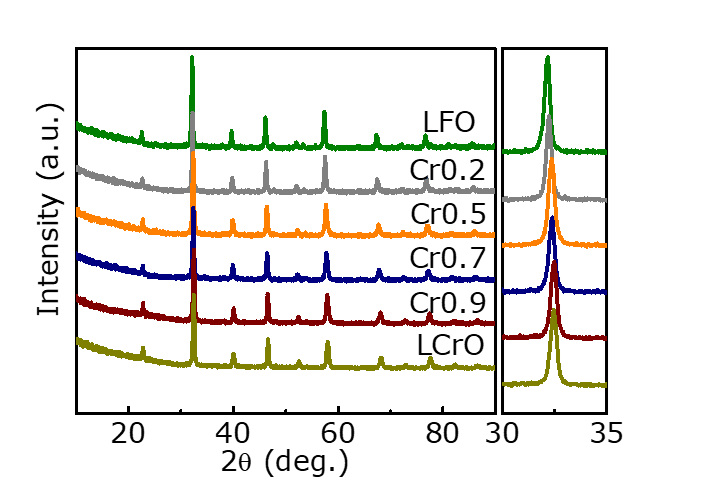


Figure S1. XRD patterns and partial magnification of LaFeO_3_, LaCr_0.5_Fe_0.5_O_3_ and LaCrO_3_


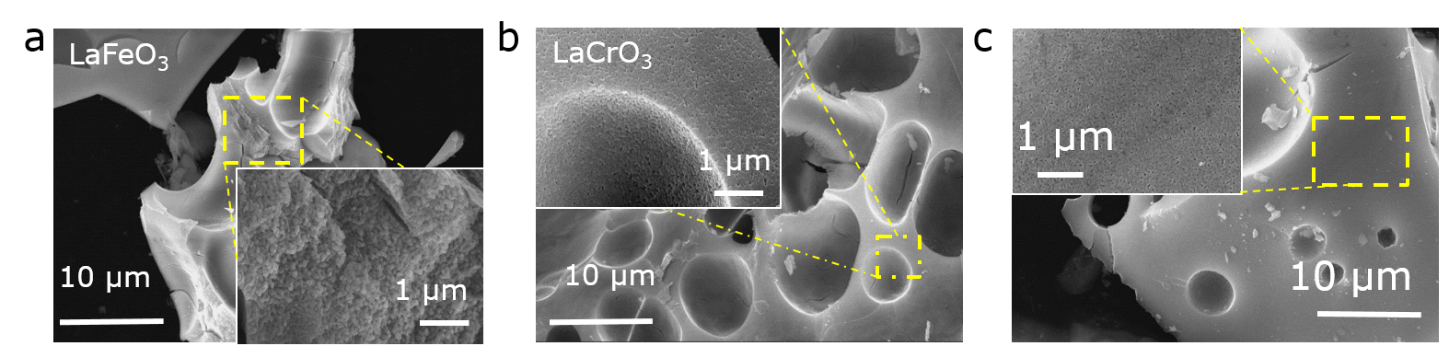


**Figure S2.** SEM images of of (a) LaFeO_3_, (b) LaCrO_3_ and (c) LaCr_0.5_Fe_0.5_O_3_.


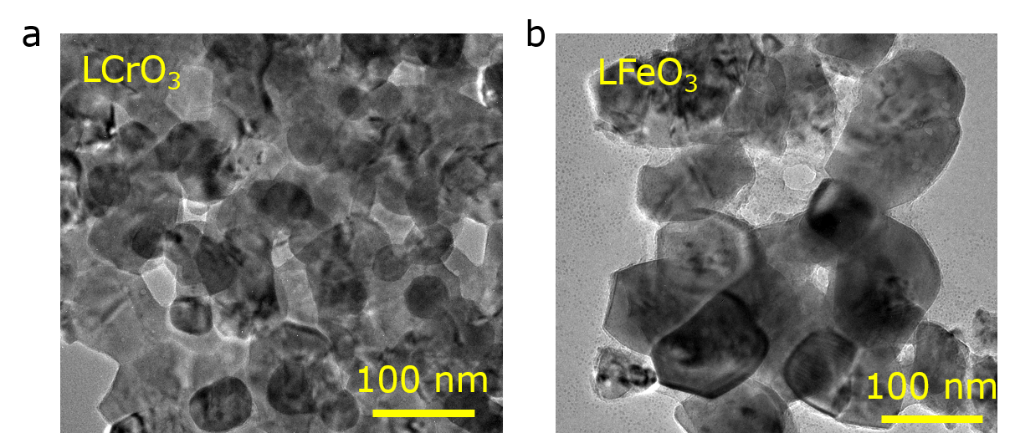


**Figure S3.** SEM images of of (a) LaCrO_3_ and (b) LaFeO_3_.


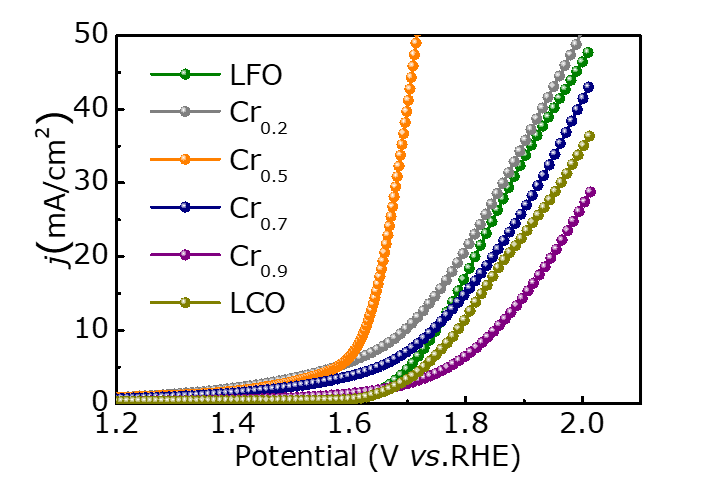


**Figure S4.** The polarization curves of all samples.


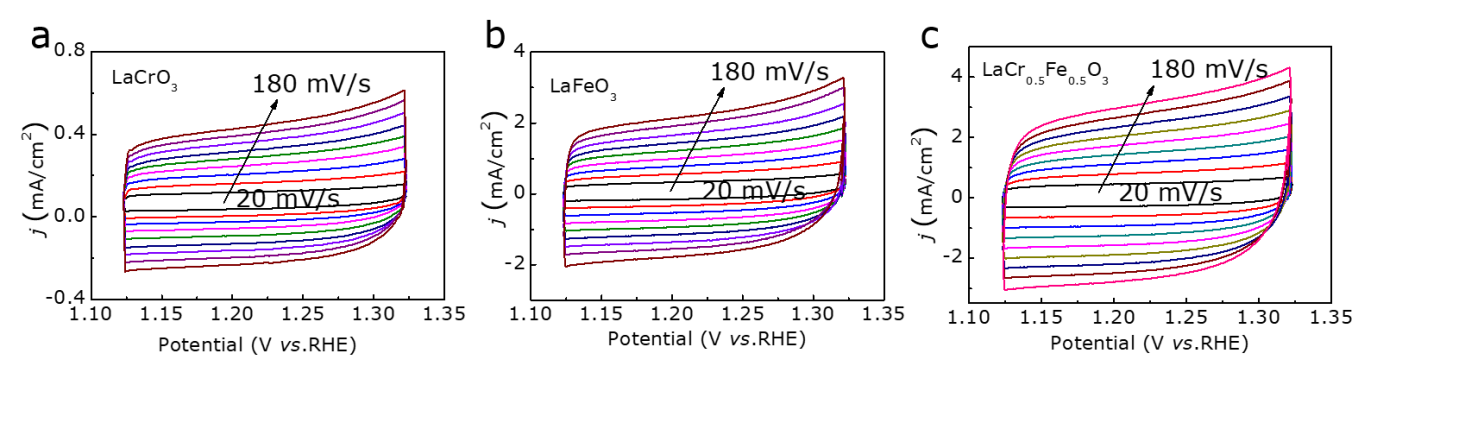


**Figure S5.** the cyclic voltammetry（CV）curve of (a) LaCrO_3_, LaFeO_3_ and LaCr_0.5_Fe_0.5_O_3_.


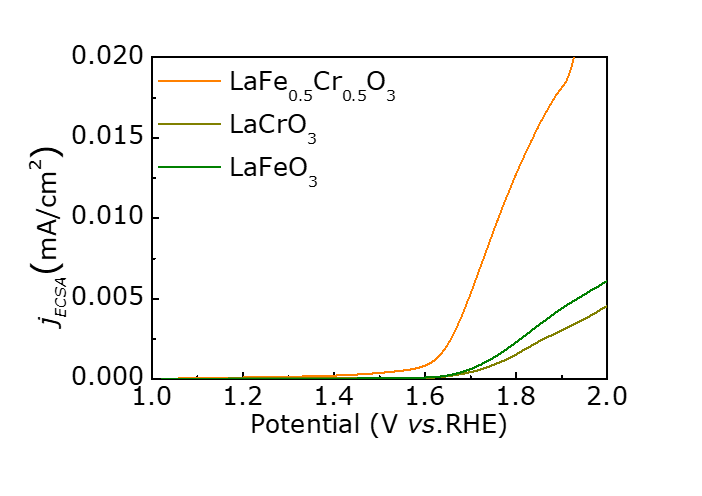


**Figure S5.** The ECSA normalized OER electrocatalytic performance.


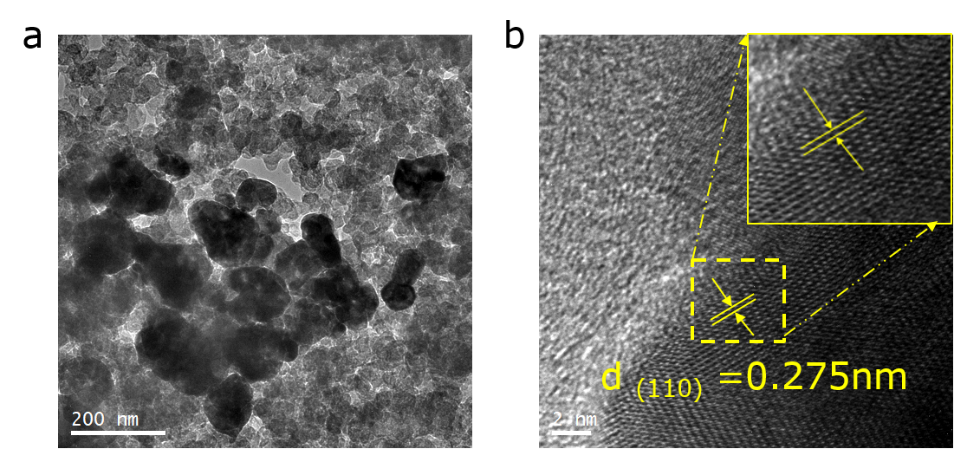


**Figure S6.** The TEM image and HRTEM image of LaCr_0.5_Fe_0.5_O_3_ after LSV test for OER.


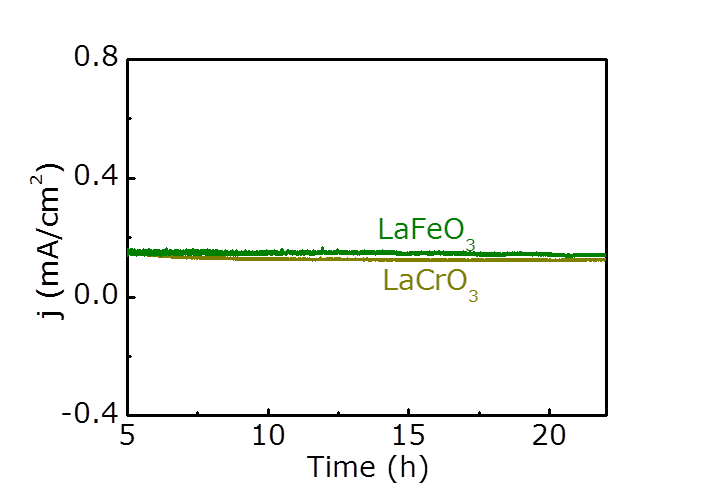


**Figure S7.** The chronoamperometry test of LaCrO_3_ and LaFeO_3_.

**Table S1.** Performance comparison of all catalysts

| catalyst | *E*_Onset_ (V) | *E_j_*_=10 mA/cm2_ |
| --- | --- | --- |
| LaFeO_3_ | 1.64 V | 1.75 V |
| LaCr_0.2_Fe_0.8_O_3_ | 1.55 V | 1.69 V |
| LaCr_0.5_Fe_0.5_O_3_ | 1.53 V | 1.62 V |
| LaCr_0.7_Fe_0.3_O_3_ | 1.57 V | 1.74 V |
| LaCr_0.9_Fe_0.1_O_3_ | 1.66 V | 1.82 V |
| LaCrO_3_ | 1.66 V | 1.78 V |

**References**

### [1] Kresse, G. & Furthmüller, J. Self-interaction correction to density functional approximation for many electron systems. *Phys. Rev. B* 54, 11169 (1996).

### [2] Kresse, G. & Joubert, D. From ultrasoft pseudopotentials to the projector augmented-wave method. *Phys. Rev. B* 59, 1758 (1999).

[3] Perdew, J. P., Burke, K. & Ernzerhof, M., Generalized Gradient Approximation Made Simple. *Phys. Rev. lett.* **77**, 3865 (1996).

[4] Rohrbach, A., Hafner, J. & Kresse, G. Molecular adsorption on the surface of strongly correlated transition-metal oxides: A case study for CO/NiO(100). *Phys. Rev. B* **69**, 075413 (2004).

[5] Rohrbach, A. & Hafner, J. Molecular adsorption of NO on NiO(100): DFT and DFT+U calculations. *Phys. Rev. B* **71**, 045405 (2005).

### [6] Cinquini, F., Giordano, L., Pacchioni, G., Ferrari, A. M., Pisani, C. & Roetti, C. Electronic structure of NiO∕Ag(100) thin films from DFT+U and hybrid functional DFT approaches. *Phys. Rev. B* 74, 165403 (2006).
